# Dental Prosthetic Treatments in Cleidocranial Dysplasia: Case Report and Literature Review

**DOI:** 10.1155/2020/8910798

**Published:** 2020-12-19

**Authors:** Yosra Mabrouk, Sinda Ammar, Amel Labidi, Lamia Mansour, Sonia Ghoul

**Affiliations:** ^1^Removable Prosthetics Department, ABCDF Laboratory (LR12ES10), Faculty of Dental Medicine, University of Monastir, Tunisia; ^2^ABCDF Laboratory (LR12ES10), Faculty of Dental Medicine, University of Monastir, Tunisia

## Abstract

Cleidocranial dysplasia (CCD) is a rare inherited skeletal syndrome. There is no consensus regarding the dental treatment strategy. *Objectives*. To report a rare case of cleidocranial dysplasia and to summarize the current clinical and dental features and prosthetic treatment of similar CCD patients reported in the literature. *Results*. A 17-year-old girl was diagnosed with CCD. She had a short stature with the ability to bring the shoulders under the chest. All remaining teeth were deciduous except the four first molars were permanent. The maxilla was hypoplastic with a relative prognathism of the mandible. The cone-beam computed tomography examination showed a distorted and incomplete root formation of the permanent teeth. She was treated with both, complete and partial, removable overdentures. PubMed was used for the literature research using the following keys words “Cleidocranial Dysplasia”[Mesh], “Prosthodontics”[Mesh], “Dental Care”[Mesh], “cleidocranial dysostosis,” and “dental treatment.” The retention of deciduous teeth was described in the majority of cases. All the patients had supernumerary teeth. The most used treatments were dental prosthetics and orthodontics. The fixed prosthetic implant was the most used type of prosthetic treatment. Among the 15 cases who specified the type of prosthetic treatment, seven patients received removable dentures. Prosthetics was indicated especially for aged patients. *Conclusion*. Removable prostheses are a good solution that rapidly restores esthetics and functions. The use of implants for these patients needs to be validated by a long-term follow-up.

## 1. Introduction

Cleidocranial dysplasia (CCD) is a rare disease that occurs in 1 per million individuals worldwide [1]. It is a disorder involving an abnormal development of bones and teeth. This dysplasia affects the entire skeleton [2] and leads to several abnormalities threatening the patient's life. Diagnosis is always based on the clinical and radiological features and could be confirmed by a genetic analysis.

The bone-related manifestations include clavicular aplasia or hypoplasia and cone-shaped thorax with short ribs. The cephalic region presents a delayed closure of sutures and fontanels with the presence of wormian bones, frontal bossing, hypertelorism, and an enlarged nose base with a depressed bridge [3].

The tooth-related manifestations are considered as major characteristics of CCD which are almost the cause of complaint. The common dental features are the retention of deciduous dentition, the presence of many supernumerary teeth, and the noneruption of permanent dentition. Other disruptions could be described such as the underdevelopment of maxilla, upward and forward mandibular rotation, and skeletal class III malocclusion tendency. Although the presence of such problems, the child patient may have no pain, no swelling problems, and no difficulties for oral functions as long as deciduous teeth are still in the mouth. Child patients do not consult at an early age. Dental disability begins later with the progressive damage of deciduous dentition. Oral deterioration becomes rapidly progressive in few years giving the patient an edentulous and aged facial appearance [4]. At that time, the majority of patients consult with a complex clinical picture.

Several therapeutic approaches have been reported in the literature including the surgical procedure combined or not with orthodontics or implant placement and the prosthodontic procedure including a removable or fixed denture. Concerning the invasive procedures, the surgical-orthodontic approach involves the extraction of unerupted supernumerary teeth, the exposure of permanent included teeth, their orthodontic traction, and their subsequent alignment [5]. The auto-transplantation of permanent teeth is another surgical alternative [5]. Concerning the implant surgical procedure, it is generally associated with the removal of deciduous and unerupted supernumerary teeth interfering with the implant placement [5]. Once osseointegration is obtained, a fixed or a removable prosthesis will be fabricated. This approach was reported since 1997 by Lombardas and Toothaker [6].

The noninvasive procedures include either fixed or removable tooth-supported prostheses. Concerning prosthetic rehabilitation with fixed prostheses, some authors propose the use of spontaneously erupted permanent teeth as abutments. However, CCD patients generally have a limited number of erupted permanent teeth. Therefore, fixed prostheses solely could not be used. Several authors [7–9] proposed the rehabilitation of this stomatognathic disorder with the removable prostheses by leaving permanent and supernumerary teeth in their positions as long as pathological changes did not occur and the use or not of deciduous teeth as abutments. This alternative is not only suitable for elderly patients, for whom orthodontic or surgical procedures could not be indicated, but also for child patients allowing them to integrate into society at an early age. In his literature review, D'Alessandro noted that the most reported cases treated with removable prostheses are children [5].

Although there is a great therapeutic choice, there is no consensus about the treatment strategy. According to Chang et al. [10], every author [11–13] almost proposes a treatment strategy that is based solely on his professional field. This ambiguity of treatment is associated with the presence of several factors in the therapeutic decision. These factors are almost related to the patients and not to the clinical findings such as their age at the time of consultation, their demands, and their treatment acceptance.

Theoretically, the surgical exposure of permanent teeth and their orthodontic traction seems to be a good option, but practically, it is a difficult and complicated treatment. In fact, after surgical extraction of supernumerary teeth and removal of the bone covering permanent teeth under general anesthesia, the prognosis of treatment remains unclear, and there is a risk for treatment failure [4]. In the past, the main treatment for these patients was prosthetic replacement [14]. That remains an interesting option that is always demanded by the patient as esthetics and function are restored rapidly and normal integration in the society is facilitated.

The need for a better understanding of this syndrome and the treatment options is necessary to better manage CCD patients. In this context, a systematic review was proposed to sum up the clinical and dental features which are almost described in CCD patients. The literature data about the treatment strategies were collected with an emphasis on prostheses. A CCD patient treated with removable prostheses was also reported.

## 2. Case Presentation

A 17-year-old girl presents at the removable prosthetics department. Her chief complaint was the unsightly retained deciduous teeth.

### 2.1. Clinical Evaluation

**Figure 1:**
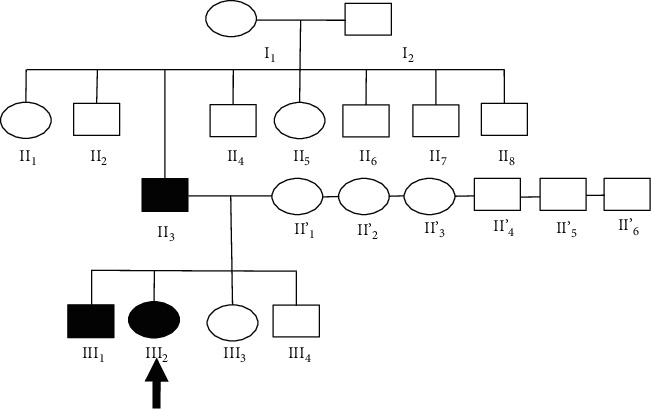
Pedigree of the studied family.

On examination, the patient had a short stature with lameness at walk. A cone-shaped thorax was noticed with an ability to bring the shoulders under the chest. The patient's father and brother also carry the same genetic defect. The transmission mode was recessive (Figure 1).

**Figure 2:**
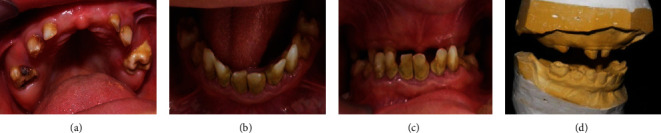
Clinical evaluation. (a) Occlusal view of maxillary arch. (b) Occlusal view of mandibular arch. (c) Intraoral photograph showing an anterior and posterior cross bite. (d) Bite registration in the correct occlusal vertical dimension.

The facial examination revealed an enlarged nose base, cheeks sagging, an unsupported upper lip, and a decreased occlusal vertical dimension (OVD). The intraoral examination showed generalized chronic periodontitis with tartar deposits in the lower anterior region. All the remaining teeth, in both the maxillary and mandibular arch, were deciduous except the four first molars were permanent. Mobility of the lower temporary incisors and canines was noticed. The upper anterior teeth were absent. The left maxillary molar was decayed (Figures 2(a) and 2(b)). The maxilla was hypoplastic with a relative prognathism of the mandible. An anterior and lateral crossbite was diagnosed (Figure 2(c)).

Casts were made and articulated at the patient's correct OVD. Registration was recorded with an interocclusal bite (Moycowax®, Aluwax®). An interocclusal rest space of 6 mm was evaluated (Figure 2(d)).

### 2.2. Radiographic Evaluation

**Figure 3:**
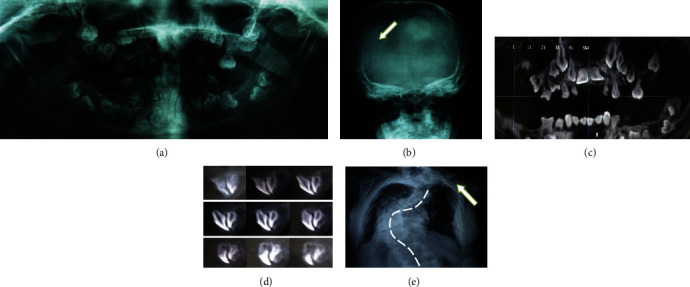
Radiographic evaluation. (a) Panoramic view of the jaws showing multiple unerupted supernumerary teeth. (b) Frontal radiograph showing a delayed closure of sagittal suture and the presence of Wormian bones. (c) Cone-beam radiograph of maxillary and mandibular arches showing a dilacerated root and inverted erupting pathway. (d) Sagittal cone-beam computed tomography section showing an incomplete formation of the left unerupted permanent incisor root. (e) Thorax radiograph showing hypoplasia of the left clavicle.

The orthopantomogram revealed the presence of many unerupted supernumeraries and permanent teeth that had not achieved their root formation, especially in the maxillary premolar regions. The second upper left molar had a suprabony situation. Only its crown was formed. The lower permanent incisors achieved their root formation. They present a right eruption pathway (Figure 3(a)). A cone-beam computed tomography examination was performed. It showed a distorted and incomplete root formation of the permanent teeth. The two definitive upper central incisors present a juxta-bone situation with a right eruption pathway. However, only its crowns were formed. The lower permanent incisors had submucosal situation (Figure 3(c) and 3(d)). The frontal radiograph showed the presence of multiple wormian bones, a widened sagittal suture, and an underdevelopment of the maxilla and paranasal sinuses (Figure 3(b)). The left clavicle was hypoplastic with a deformation of the vertebral column in the thorax X-ray (Figure 3(e)).

### 2.3. Ultrastructural Evaluation

**Figure 4:**
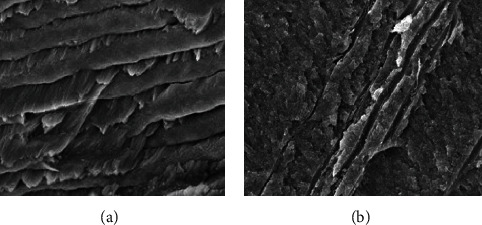
Ultrastructural evaluation. (a) A scanning electron microscopy (zoom ×2000) view showing the prismatic aspect of the enamel of the patient's temporary tooth. (b) A scanning electron microscopy (zoom ×2000) view showing some dentinal tubules in longitudinal section.

Upon a scanning electron microscopic examination, the extruded deciduous teeth showed normal structures either for the enamel, dentine, or pulp (Figures 4(a) and 4(b)).

### 2.4. Dental Treatment

**Figure 5:**
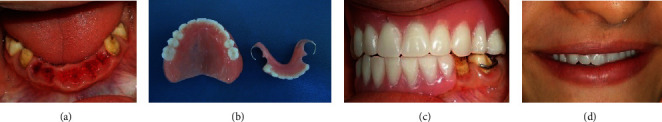
Dental treatment. (a) Extraction of the mandibular incisors and canines. (b) Interim maxillary and mandibular prostheses. (c) Prostheses adjusted in the mouth. (d) Final clinical view.

After obtaining the patient's consent, the lower incisors and canines were extracted (Figure 5(a)). The patient had an incomplete root development of her permanent unerupted teeth with a generalized chronic periodontitis that did not allow orthodontic intervention. The treatment was oriented to the prosthodontic option. The patient had one permanent tooth in each hemiarch. Moreover, there was an important interocclusal rest space. These conditions could not allow the exploitation of the remaining permanent teeth as abutments for fixed prostheses. The use of implants was also eliminated because the patient refused a surgical extraction of the supernumerary teeth in the implant site. In addition, there was a risk of bone fragility given the high number of supernumerary and unerupted teeth to be extracted.

The decision was to make a complete removable overdenture at the maxilla and a partial removable denture at the mandible. The deciduous teeth were used as abutments for a complete prosthesis.

Individual trays were fabricated using diagnostic casts. Master impressions were recorded in polysulfide material (Surflex®). Occlusal registration was performed at the correct reestablished OVD. The mounted prosthetic teeth were checked and approved by the patient. The two removable prostheses were polymerized, fitted, and adjusted in the patient's mouth. Retention and stability of prostheses were excellent. The patient was very pleased and satisfied with her new appearance (Figure 3(b) and 3(c)).

The patient consulted every 2 months for a check-up. She has been followed for 4 years. The deciduous canines with mobility were extracted, and the prostheses were relined. Although the primary lower incisors and canines were avulsed, the corresponding permanent teeth did not erupt.

## 3. Literature Review

### 3.1. Statement of the Problem

Cleidocranial dysplasia is a rare inherited skeletal syndrome. It affects especially the clavicular bone and teeth. Several treatment options are used including orthodontics, surgery, and prosthodontics, but there is no consensus regarding the treatment strategy. The prosthodontic alternative remains a simple, cheap, and rapid solution.

### 3.2. Search Strategy

The explored database was Medline using the PubMed interface. The search strategy was conducted using the terms “Cleidocranial Dysplasia”[Mesh], “Prosthodontics”[Mesh], “Dental Care”[Mesh], “cleidocranial dysostosis,” and “dental treatment.” The review focused on combinations of these terms to collect the maximum of articles related to the subject. Four Boolean formula were made: (“Cleidocranial Dysplasia”[Mesh]) AND “Prosthodontics”[Mesh]), (“cleidocranial dysostosis” AND “Prosthodontics”[Mesh]), (“Cleidocranial Dysplasia”[Mesh]) AND “Dental Care”[Mesh]), and (“Cleidocranial Dysplasia”[Mesh]) AND “dental treatment”). The final search update was in June 2016.

The exclusion criteria included the date of publication (<1996), and only the articles written in French or English were retained. All types of articles were included except reviews. The data described by the authors in these papers were collected and analyzed by five reviewers independently using a preestablished checklist for data extraction (clinical photographs or radiographs were not used to include additional findings). In case of disagreement, a consensus was obtained by discussion among the reviewers.

### 3.3. Results

#### 3.3.1. Article Selection

**Figure 6:**
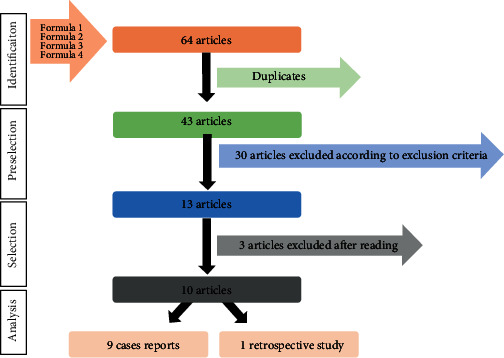
Articles selection.

Sixty-four articles were obtained after the search. Considering the exclusion criteria and duplicates, 13 articles were retained. Three papers were excluded after reading (Figure 6). One article was not available.

#### 3.3.2. Clinical Characteristics and Dental Features of the Patients That Were Included in This Systematic Review (Tables 1 and 2)

Nine papers were included in the systematic review. Among these papers, eight concerned 10 case reports and 1 retrospective study described 15 patients. A total of 25 patients were included in the study, the youngest was 8 years old, and the oldest was 46. Twelve of them were male, and 13 were female. Twenty-one were in mixed dentition while four of them were in permanent dentition. Clavicular aplasia was noticed in 19 cases and hypoplasia in two cases while clavicular abnormalities were not specified in 4 cases. Delayed fontanel ossification was described in 15 patients. Inheritance was described in one case as autosomal dominant.

The dental features described in these patients covered retention of deciduous teeth (8 patients), unerupted permanent teeth (10 patients), dental shape abnormalities (3 patients), and skeletal class III (2 patients). All the patients showed supernumerary teeth while dental structure abnormalities were not specified in any case.

#### 3.3.3. Dental Therapies of the Patients That Were Included in This Systematic Review (Table 3)

Seven treatment options were described in this review. The most used ones were the prosthetic (all patients) and orthodontic alternatives (22 patients). Periodontal surgery (16 patients) and conservative odontology (17 patients) were also frequently used. Extraction of supernumerary teeth was associated with treatment in 9 cases. The patients' follow-up was mentioned in three cases varying from 1 to five years.

#### 3.3.4. Prosthetic Treatment (Table 4)

The type of prosthetic treatment was not specified for the 15 patients of the retrospective study. Among the 10 other patients, 5 received implant fixed prosthetics (only one case described the treatment follow-up), 4 patients had removable partial prostheses, 3 patients were treated with complete prostheses, one patient fixed prosthesis, and one had patient implant-supported removable complete prostheses. No overdenture was described.

Twenty-two patients among the 25 received a combination of orthodontic and prosthodontic treatment. Concerning the chronology of these combined treatments, for the 15 patients included in the retrospective study, the author did not mention whether he started with the orthodontic or prosthodontic treatment. However, orthodontic treatment prior to prosthodontic treatment was described in 5 cases. Among these cases, orthodontic treatment was stopped or failed in 3 patients. In 2 cases, orthodontic treatment was achieved followed by prosthetic implants. All the patients who received an orthodontic treatment were lastly treated with prostheses. Three patients received only prostheses, the first case was treated with removable complete prostheses and implant-supported removable complete prostheses, the second with removable partial prostheses in the two dental arches, and the third with a fixed prosthetic implant.

## 4. Discussion

CCD patients present many challenges in diagnostics as well as in the treatment.

For diagnostics, the difficulty lies in the variability of the clinical and radiological manifestations of this syndrome. As it is described in the illustrated case, the common features are short stature, narrow chest with hypermobility of the shoulders, and dental disorders involving retention of the deciduous teeth, delayed eruption of permanent teeth, and the presence of many supernumerary teeth. Diagnosis could be confirmed by a genetic analysis. In fact, this condition is usually caused by a mutation of the RUNX2 gene located at chromosome 6p21. The difficulty also lies in the presence of other diseases having similar features such as crane-Heise syndrome, mandibuloacral dysplasia, pycnodysostosis, and Yanis-Varon syndrome [15]. Histopathological features are not frequently reported, but the findings are controversial in the literature. In fact, in the present case, there were no perturbations in the tooth structure. However, Vij et al. [16], in his histological analysis, found distorted dentinal tubules, prominent interglobular dentine, and acellular cementum with the absence of cellular cementum in the apical region. Yet, Lukinmaa et al. found a regular dentine structure in his observation [17].

The rarity of CCD syndrome makes the guidelines for its treatment scanty in the literature. So it is important to present any treated cases [18]. In this review, the majority of the published papers are case reports. In these lasts, the treatment philosophy was not the same and seemed to be based on the authors' experiences.

Planning treatment is difficult. A whole set of questions must be answered concerning the supernumerary teeth, removing them surgically or intervening only if pathological changes occur; concerning the remaining deciduous teeth, leaving them until they exfoliate naturally or removing them with the purpose of facilitating eruption of the permanent teeth into the arch; and concerning the age of intervention [2].

The literature analysis highlights the variety of therapeutic choices. They can involve orthodontics, surgery, or prosthodontics.

The surgical exposure of permanent teeth associated with orthodontic traction at an early age is an interesting solution. In fact, the patient's natural dentition will be spared and a good function and esthetics will be achieved. According to Kargul [19], the surgical exposure of unerupted teeth can lead to cementum formation and eruption of a dentition with normal root formation at an early age. The obvious disadvantage of this approach is the extensive duration of treatment, requiring multiple surgical procedures which could be expensive for the patient and challenging for the practitioner [20]. Several dental problems could not be resolved with this treatment option such as irregular dentition due to shape abnormalities. Besides, calcification and the loss of some teeth due to the presence of caries after orthodontic treatment are mentioned in the literature. Added to that, orthodontic treatment can be interrupted or failed. In fact, in the present review, among the 22 patients who had received orthodontic treatment, there were 3 cases for whom the treatment was stopped by the patients or failed, and several cases were described without follow-up.

The prosthodontic treatment was the most used alternative as all the patients included in this review were treated with prostheses. Chang et al. [10] thought that without prosthodontic treatment, occlusion and esthetics do not improve. In fact, in this review, the 2 cases that had a successful orthodontic treatment received a prosthodontic treatment. The majority of patients who were treated with only prostheses were aged more than 15 years. This treatment was indicated for aged patients for whom the displacement of teeth with orthodontics was difficult. Among the 10 patients, five had received implants. This alternative seemed to be an interesting option that provides a good result in a short time. The use of implants for CCD patients is controversial in the literature. In fact, according to Butterworth [3], the use of endosteal implants is generally contraindicated due to the presence of multiple unerupted teeth which reduce the amount of available bone. Petropoulos et al. [21] described a case of a CCD patient using 16 osseointegrated implants. After extraction of all the teeth, an alveoloplasty was carried out. The harvested bone was then put into the osseous defects. The entire treatment lasted 11 months. However, a long-term follow-up was needed to confirm the validity of this treatment and the quality of osseointegration of these implants regarding bone defects. In fact, the incriminated gene is not only expressed in the teeth but also in the bone. While bone abnormalities are described microscopically, little is known about the biological features of this tissue, particularly during osseointegration.

The placement of the implant through impacted teeth to avoid invasive surgery should be considered. While this option was described in the literature [22, 23], it was never described for CCD patients. More studies are needed before this unconventional procedure might be considered as a possible clinical option.

Removable prostheses and overdentures were used in five patients. They can be considered as a rapid solution that restores esthetics and functions. Deciduous teeth can be used as abutments for these dentures. But several questions can be raised whether they are able to withstand the forces applied to them when the prosthesis is in function or they will resorb prematurely [3]. The extraction of these teeth does not necessarily induce the eruption of permanent teeth [24]. So, these teeth could be preserved if removable prostheses were indicated. The prosthesis should be relined whenever some of these teeth are lost. For the presented case, the patient refused a long and surgical treatment. Removable prostheses were provided, and the patient was very pleased with the result.

## 5. Conclusion

The dental treatment of CCD patients is complex. Practitioners should be aware of the different therapeutic options to indicate the appropriate one according to the patients' age, their demands, and their compliance with the treatment. At an early age, a long treatment such as surgical-orthodontic approach can be proposed. But at late age, a rapid solution is almost used such as the prosthetic treatment. The removable denture offers a good result, but retention depends on the number of teeth remaining in the arch. Implants are frequently used. However, there is a need for clinical long-term studies to confirm the validity of the treatment.

## Figures and Tables

**Table 1:** Clinical characteristics of the patients included in the systematic review.

No.	Authors	Study design	Age (years)	Sex	Dentition	Clavicular abnormalities	Delayed fontanels ossification	Inheritance
1 [10]	Chang et al.	RS	(9-46)	8 M, 7 F	Mx	15/15 aplasia	14/15 patients	Ns
2 [25]	Petropoulos et al.	CR (1)	45	F	P	Aplasia	Ns	Ns
3 [26]	Berg et al.	CR (1) (2)	13	M	Mx	Ns	Ns	Ns
			13	F	Mx	Aplasia	Ns	Ns
4 [13]	Olszewska	CR (1)	40	M	P	Ns	Ns	Ns
5 [20]	Daskalogiannakis et al.	CR (1) (2)	39	F	P	Ns	Ns	Ns
			8	M	Mx	Ns	Ns	Ns
6 [27]	Angle and Rebellato	CR (1)	10	F	Mx	Aplasia	Ns	Ns
7 [21]	Petropoulos et al.	CR (1)	42	F	P	Hypoplasia	Ns	Ns
8 [3]	Butterworth	CR (1)	9	M	Mx	Aplasia	Ns	Ns
9 [19]	Kargul et al.	CR (1)	12	F	Mx	Hypoplasia	Yes	Autosomal dominant

RS: retrospective study; CR: case report; F: female; M: male; P: permanent dentition; Mx: mixed dentition; Ns: not specified; FB: frontal bossing; FD: frontal depression.

**Table 2:** Dental features of the patients included in the systematic review.

No.	Retention of deciduous teeth	Unerupted permanent teeth	Shape abnormalities	Structure abnormalities	Skeletal class	Supernumerary teeth
1 [10]	Ns	Ns	Ns	Ns	Ns	12 yes
2 [25]	No	Yes	Yes^1^	Ns	Ns ^2^	Yes
3 [26]	Yes	Yes	Ns	Ns	Ns	Yes
	Yes	Yes	Ns	Ns	Ns	Yes
4 [13]	Yes	Yes	Ns	Ns	Cl III	Yes
5 [20]	No	Yes	Ns	Ns	Ns	Yes
	Yes	Yes	Ns	Ns	Ns	Yes
6 [27]	Yes	Yes	Yes	Ns	Cl III	Yes
7 [21]	Yes	Yes	Ns	Ns	Ns	Yes
8 [3]	Yes	Yes	Yes	Ns	Ns	Yes
9 [19]	Yes	Yes	Ns	Ns	Ns	Yes

^1^Malformation and short conical roots. ^2^Dental class I and anterior cross bite. Ns: not specified; Cl III: skeletal class III malocclusion.

**Table 3:** Dental therapies of the patients included in the systematic review.

No.	Orthodontic treatment	Prosthetic treatment	Orthognathic surgery	Periodontal surgery	Conductive osteotomy	Conservative odontology	Extraction of supernumerary teeth	Patients' follow-up (year)
1 [10]	+	+	-	+	+	+	-	Ns
2 [25]	+∗^1^	+^2^	Ns	Ns	-	-	+	5
3 [26]	+^3^	+^3,2^	+	-	-	-	+	Ns
	+^3^	+^3^	-	-	-	-	+	
4 [13]	+∗^1^	+^2^	-	-	+	-	+	Ns
5 [20]	(1)-	+	-	-	-	-	+	4
	(2)+^1^	+^2^	+	-	+	-	-	Ns
6 [27]	+^1^	+^2^	-	+	+	+	+	Ns
7 [21]	-	+	-	-	+	-	+	Ns
8 [3]	+∗^1^	+^2^	-	-	+	+	+	1
9 [19]	-	+	-	-	-	-	+	Ns

^1^First-line treatment. ^2^Second-line treatment. ^3^Simultaneous treatment. ^∗^Failure or interruption of the treatment. Ns: not specified.

**Table 4:** Prosthetic treatment.

No.	Implant prosthetics	Complete prosthesis	Removable partial prosthesis	Fixed prosthesis	Over denture	Implant-supported removable complete prosthesis	Implant-supported removable partial prosthesis	Hybrid prosthesis
1 [10]	Ns	Ns	Ns	Ns	Ns	Ns	Ns	Ns
2 [25]	+	+^1^	-	-	-	-	-	-
3 [26]	(1) + (2)-	- -	+ +	- -	- -	- -	- -	- -
4 [13]	Ns	Ns	Ns	Ns	Ns	Ns	Ns	Ns
5 [20]	(1)- (2)+	+ -	- -	- -	- -	+ -	- -	- -
6 [27]	+	-	-	-	-	-	-	-
7 [21]	+	+	-	-	-	-	-	-
8 [3]	-	-	+	+	-	-	-	-
9 [19]	-	-	+	-	-	-	-	-

^1^Transitional complete prostheses after implant surgery. Ns: not specified.
